# Hydrocortisone decreases lethality and inflammatory cytokine and nitric oxide production in rats challenged with *B. anthracis* cell wall peptidoglycan

**DOI:** 10.1186/s40635-020-00358-4

**Published:** 2020-11-18

**Authors:** Yan Li, Xizhong Cui, Joseph Shiloach, Jeffrey Wang, Dante A. Suffredini, Wanying Xu, Wancang Liu, Yvonne Fitz, Junfeng Sun, Peter Q. Eichacker

**Affiliations:** 1 Critical Care Medicine Department, NIH Clinical Center, National Institutes of Health, Building 10, Room 2C145, 10 Center Drive, Bethesda, MD 20892 USA; 2 Biotechnology Core Laboratory, National Institute of Diabetes, Digestive, and Kidney Diseases, National Institutes of Health, Bethesda, MD 20892 USA; 3 Section of Critical Care, Department of Medicine, St. Agnes Hospital, Baltimore, MD 21229 USA

**Keywords:** *B. anthracis*, Shock, Cell wall, Peptidoglycan, Anti-inflammatory agents, Corticosteroids, TNF-soluble receptor

## Abstract

**Background:**

Lethal *B. anthracis* infection produces high proinflammatory peptidoglycan (PGN) burdens in hosts. We investigated whether the lethality and inflammation anthrax PGN can produce are related.

**Methods:**

At 6 h before and the start of 24 h anthrax PGN infusions, rats (n = 198) were treated with diluent (controls) or one of three IV-doses of either hydrocortisone (125, 12.5 or 1.25 mg/kg) or TNF-soluble receptor (TNFsr; 2000, 1000 or 333 μg/kg), non-selective and selective anti-inflammatory agents, respectively.

**Results:**

Compared to controls, hydrocortisone 125 and 12.5 mg/kg each decreased 7-day lethality (*p* ≤ 0.004). Hydrocortisone 125 mg/kg decreased IL-1β, IL-6, TNFα, MCP, MIP-1α, MIP-2, RANTES and nitric oxide (NO) blood levels at 4 and 24 h after starting PGN (except MCP at 24 h). Each decrease was significant at 4 h (except MIP-1α that was significant at 24 h) (*p* ≤ 0.05). Similarly, hydrocortisone 12.5 mg/kg decreased each measure at 4, 24 and 48 h (except TNFα at 24 h and MIP-1α at 24 and 48 h and NO at 48 h). Decreases were significant for IL-6 and NO at 4 h and RANTES at 48 h (*p* ≤ 0.05). Hydrocortisone 1.25 mg/kg had non-significant effects. Each TNFsr dose decreased lethality but non-significantly. However, when doses were analyzed together, TNFsr decreased lethality in a potential trend (*p* = 0.16) and IL-6 and NO significantly at 4 h (*p* = 0.05).

**Conclusions:**

Peptidoglycan-stimulated host inflammation may contribute to *B. anthracis* lethality.

## Introduction

Outbreaks and isolated cases of *Bacillus anthracis* (*B. anthracis*) infection in the United States and Europe over the past 15 years and this bacterium’s weaponization potential, have raised persistent concerns [1–3]. Invasive forms of infection produce shock resistant to supportive measures and have a poor prognosis [3–5]. Anthrax lethal and edema toxin (LT and ET) have long been associated with *B. anthracis* lethality. Lethal toxin is a potent inhibitor of stress kinase pathways while ET has adenyl cyclase activity that increases intracellular cAMP to very high levels [3]. Both have the ability to produce shock and organ injury in animal models [6]. Notably though, shock and lethality with LT and ET have been shown not to be associated with the kind of systemic inflammatory response associated with bacterial sepsis [7, 8]. However, increasing evidence suggests that the *B. anthracis* cell wall and its peptidoglycan (PGN) component may contribute to shock and lethality with this bacterium, in part by stimulating maladaptive inflammatory responses [5, 9]. Infusion of viable lethal *B. anthracis* in baboons produced increases in inflammatory cytokines, shock and organ injury [10]. We then showed that *B. anthracis* cell wall alone produced a similar inflammatory response and lethality in rats [11]. In a subsequent study we conducted comparing similarly lethal 24-h challenges of *B. anthracis* LT, ET or purified cell wall PGN in rats, only PGN produced significant increases in circulating inflammatory cytokine and nitric oxide levels and coagulopathy consistent with disseminated intravascular coagulation (DIC) [12]. The pattern and time course of these coagulopathic changes with anthrax PGN challenge were also noted in a baboon model by another group that has studied this component’s inflammatory effects [13–16]. Although excessive inflammation is implicated in the pathogenesis of cell wall components from other bacteria, it may be more important during *B. anthracis* infection which produces exceptionally high bacteria burdens in blood and tissue and provides a potentially large PGN reservoir [17–19].

While it is clear *B. anthracis* PGN stimulates a host inflammatory response, whether this proinflammatory effect contributes to lethality and organ injury is unknown. To examine this question, we pre-treated Sprague-Dawley rats with high, medium or low doses of either hydrocortisone (HC), a nonselective anti-inflammatory agent, or tumor necrosis factor soluble receptor (TNFsr), a selective one. Rats were then challenged with 24-h anthrax PGN infusions to simulate its release during bacterial infection. We hypothesized that suppressing host inflammation, either non-selectively with HC or selectively with TNFsr, would reduce PGN-associated lethality, inflammatory cytokine levels, and organ injury.

## Methods

### Animal care

This study was approved by the Animal Care and Use Committee of the Clinical Center of the National Institutes of Health, protocol #ASP CCM 1801.

### Study design

Nineteen weekly experiments were performed examining either hydrocortisone (HC) or TNFsr (Additional file 1: Table S1). Male Sprague-Dawley rats (*n* = 198 total) weighing 250 to 300 g with indwelling carotid arterial and jugular venous catheters were challenged with an LD40 to LD80 dose of *B. anthracis* PGN infused over 24 h via the venous catheter. Three sets of weekly experiments examined either high, medium or low doses of HC (125, 12.5 or 1.25 mg/kg, respectively) and three other sets examined high, medium or low doses of TNFsr (2000, 1000 or 333 μg/kg, respectively). In each weekly experiment, animals were randomized to receive the dose of anti-inflammatory agent being studied (*n* = 4 to 6) or diluent (control, *n* = 4 to 6) administered 6 h before (T-6) and at the time the PGN infusion was started (T0). From 6 h before until 48 h following the start of the PGN infusion, animals had mean arterial blood pressure (MBP) and heart rate (HR) continuously measured and data at 3 h intervals was analyzed. At 4, 24 and 48 h after the start of PGN, 0.5 ml blood was drawn from the arterial catheter for measurement of seven cytokines (IL-1β, IL-6, TNFα, MIP-1α, MIP-2, MCP-1 and RANTES), nitric oxide (NO), complete blood count (CBC), arterial blood gas (ABG) with lactate, and chemistries with Na, K, Cl, alanine and aspartate amino-transferases (ALT and AST), creatine phosphokinase (CK) and blood urea nitrogen (BUN) and creatinine (Cr). Sampled blood was replaced with saline 0.5 ml. Animals alive following 168 h of observation were considered survivors and were euthanized. This study was initially designed to only include hemodynamic measures for 24 h. But after experiments testing the hydrocortisone dose 125 mg/kg were completed, a decision was made to extend these measures until 48 h.

### Peptidoglycan and treatment preparation and dosing

Peptidoglycan was isolated and prepared from *B. anthracis* strain ΔSterne strain, which lacks capsule and toxins, as previously described [14, 16]. Briefly, bacteria grown overnight on tryptic soy broth plates were boiled in 8% SDS for 30 min and centrifuged. The pellet was washed with endotoxin-free water and subjected to DNase I and RNase A treatment. The sample was boiled in 4% SDS for 30 min and washed three times with endotoxin-free water. The pellet was then treated with NaCl 2 M, washed six times with endotoxin-free water, dried, weighed, resuspended in endotoxin-free water and treated with hydrofluoric acid (HF) to remove the PGN-associated polysaccharide [20]. Following HF treatment, the PGN was treated with a denaturing buffer [50 mM Tris (pH 8.0), 6 M guanidine HCl, 25 mM dithiothreitol (DTT)] at 60 °C for 1 h. Iodoacetamide 75 mM was added and the preparation was incubated for 15 min in the dark to alkylate Cys residues. The reaction was stopped with DTT 40 mM. The PGN was resuspended in a buffer containing 50 mM Tris (pH 7.5), 1 M guanidine HCl, and 5 mM CaCl_2_ and was treated with 20 μg proteinase K, added every 12 h for 36 h at 50 °C. Finally, the PGN was washed three times with endotoxin-free water, dried, weighed, and resuspended in endotoxin-free water when used. It required 1.7 × 10^10^ CFU to produce 1 mg of purified PGN. The methods were previously shown to produce preparations free of other PGN binding molecules [12].

Three separate batches of PGN were employed for each dose of hydrocortisone investigated (i.e., one batch for each dose) and one batch for all TNFsr doses studied. Following preparation of each PGN batch, a survival dose response study was performed, and the PGN dose producing a lethality rate of 40 to 80% was employed in subsequent experiments. This dose was found to be 80 mg/kg administered at 3.3 mg/kg/h for 24 h for all experiments. As determined by the chromogenic limulus amoebocyte lysate assay (Clonogen, Germantown, MD) the lipopolysaccharide (LPS) content for each PGN preparation was ≤ 1.9 ng/mg and the LPS amount administered during a 24 h infusion of PGN for the average size animal studied was ≤ 152 ng/kg. Compared to diluent control rats (*n* = 6), a 24 h LPS infusion of 160 ng/kg in animals (*n* = 6) produced no lethality and did not significantly increase IL-1β, IL-6, TNFα, MIP-1α, MIP-2, MCP-1, RANTES or NO at 4 or 24 h after the start of challenge (except MIP1α at 4 h, *p* = 0.007). By contrast, compared to another group of diluent controls (*n* = 14), a 24 h PGN infusion of 80 mg/kg (*n* = 12) produced 50% lethality and significant increases in each of these parameters at 4 h (*p* = 0.005) and in IL-1β, MCP-1, MIP-1α, MIP-2 and NO at 24 h (*p* ≤ 0.009).

Based on a factor of 0.162 to convert animal to human dosing, the HC 125, 12.5 or 1.25 mg/kg doses administered at T-6 and T0 in rats in these experiments would be equivalent to total doses in a 70 kg human of 2800, 280, and 28 mg [21]. These doses are comparable to a pulse, stress or maintenance HC dose, respectively, administered clinically [22–25]. The doses of TNFsr investigated here in rats (2000, 1000 and 333 μg/kg administered at T-6 and T0) would be equivalent to total doses in a 70 kg human of 45.4, 22.7, and 7.4 mg. The two higher TNFsr doses studied are equivalent to the 50 and 25 mg doses recommended for humans with rheumatologic disease [26, 27]. The dose of 333 μg/kg employed here was greater than a dose of 250 μg/kg we previously showed was protective in rats challenged with lethal intravenous and intrabronchial *E. coli* challenges [28].

### Laboratory measures

As previously described, protected catheters were attached to exteriorized arterial and central venous access ports on each animal [7]. Central venous catheters were attached via 3-way stop-cocks to a syringe pump to provide PGN as an infusion. Arterial catheters were connected to transducers to determine arterial blood pressure and heart rates. Arterial blood was collected for CBC, ABG and lactate, chemistry, cytokine and nitric oxide measures. Cytokine (IL-1β, IL-6, TNFα, MIP-1α, MIP-2, MCP-1 and RANTES) and NO levels and CBC and ABG were determined as previously described [12, 28].

### Statistics

Survival times were plotted using Kaplan–Meier survival curves and analyzed using stratified log-rank tests and Cox proportional hazard model. For all other variables, we used linear mixed models to account for repeated measurements of each animal and the actual pairing of animals within each cycle. Standard residual diagnostics were used to check model assumptions. Due to limitations in the amount of purified PGN and animals available for study, the numbers of animals investigated with doses of either HC or TNFsr was based on whether there was a trend in efficacy noted in early experiments with each dose and the amount of PGN and animals available for study. Data were log-transformed when needed. SAS version 9.4 (Cary, NC) was used for all analyses. All p-values are two-sided.

## Results

### Effects of hydrocortisone

**Fig. 1:**
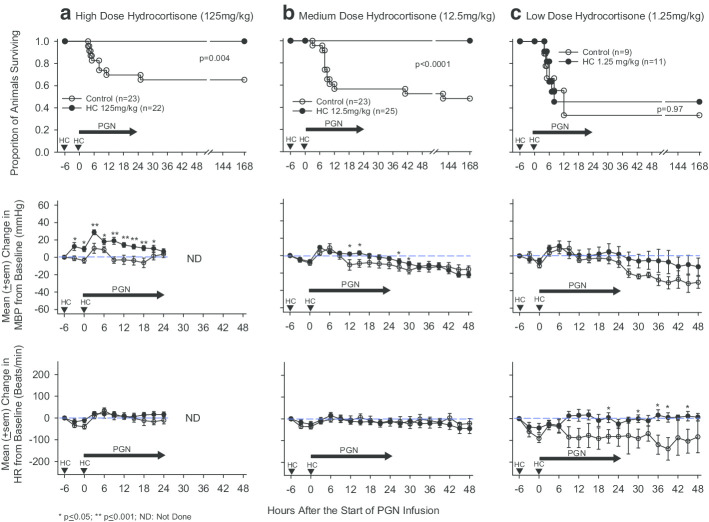
This figure compares proportional survival and mean (± sem) serial changes in mean arterial blood pressure (MBP) and heart rate (HR) during and after a 24-h *B. anthracis* peptidoglycan infusion (PGN, horizontal arrows) in diluent (control) treated animals versus animals treated with either a high (125 mg/kg) (**a**), medium (12.5 mg/kg) (**b**) or low (1.25 mg/kg) (**c**) dose of hydrocortisone (HC). Hydrocortisone or diluent treatment were administered intravenously 6 h before and at the start of PGN infusion (inverted dark arrow heads). Survival is shown from the start of PGN and serial changes in MBP and HR were measured and shown from 6 h before until either 24 h (high-dose HC) or 48 h (medium and low-dose HC) after the start of PGN

Compared to diluent controls, following initiation of PGN challenge, survival with hydrocortisone was increased significantly with the high (125 mg/kg) and medium (12.5 mg/kg) HC doses [15 survivors of 23 total controls (65.2% survival) vs. 22 of 22 HC 125 mg/kg animals (100% survival), *p* = 0.004; 11 of 23 controls (47.8% survival) vs. 25 of 25 HC 12.5 mg/kg animals (100% survival), *p* < 0.0001], but not with the lowest dose (1.25 mg/kg) [3 of 9 controls (33.3% survival) vs. 5 of 11 HC animals (45.5% survival), *p* = 0.97] (Fig. 1). Compared to controls, high-dose HC increased mean arterial blood pressure (MBP) from 3 h after its first administration until 21 h after the start of the PGN infusion (*p* ≤ 0.05). Medium-dose HC increased MBP at 12, 15, 27 and 30 h after the start of PGN infusion (*p* ≤ 0.05). Low-dose HC did not alter MBP significantly (p > 0.05 for all measures). Neither high nor medium-dose HC altered heart rate (HR) significantly although low-dose HC increased HR at 21, 30, 36, 39, and 45 h after the start of PGN (*p* ≤ 0.05).

**Fig. 2:**
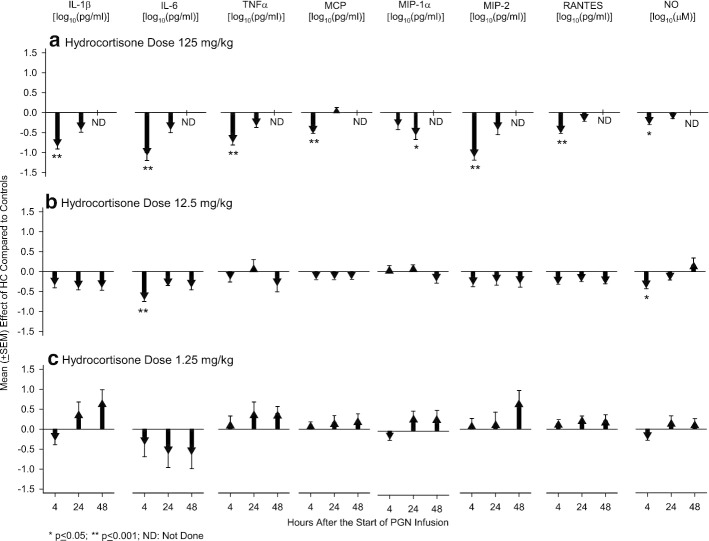
This figure shows the mean effect (± SEM) of either a high (125 mg/kg) (**a**), medium (12.5 mg/kg) (**b**) or low (1.25 mg/kg) (**c**) dose of hydrocortisone (HC) compared to diluent (controls) on blood levels of IL-1β, IL-6, TNFα, MCP, MIP-1α, MIP-2, RANTES [all log_10_(pg/ml)] and nitric oxide [NO, log_10_(μM)] at 4 and 24 h for high-dose hydrocortisone and 4, 24 and 48 h for medium and low doses following the start of a 24 h peptidoglycan infusion. Down and upgoing going arrows indicate the amount that hydrocortisone decreased or increased, respectively, the parameter measured compared to control

High and medium but not low-dose HC suppressed the intravascular inflammatory response stimulated by PGN. Compared to controls, after the start of PGN infusion, high-dose HC decreased all cytokines and NO at both 4 and 24 h (except for MCP-1 at 24 h) and these decreases were significant for IL-1β, IL-6, TNFα, MCP-1, MIP-2, RANTES and NO at 4 h and for MIP-1α at 24 h (*p* ≤ 0.05) (Fig. 2, Additional file 1: Table S2). In a similar pattern, but less significantly, medium-dose HC decreased all cytokines and NO at 4, 24 and 48 h except for TNFα at 24 h, MIP-1α at 4 and 24 h and NO at 48 h. These decreases in IL-6 and NO with medium-dose HC were significant at 4 h (*p* ≤ 0.05). Different from high and medium-dose HC, low-dose HC was associated with non-significant increases in cytokines and NO levels at most time points except for IL-1β, MIP-1α and NO at 4 h and IL-6 at the three time points that had non-significant decreases.

**Fig. 3:**
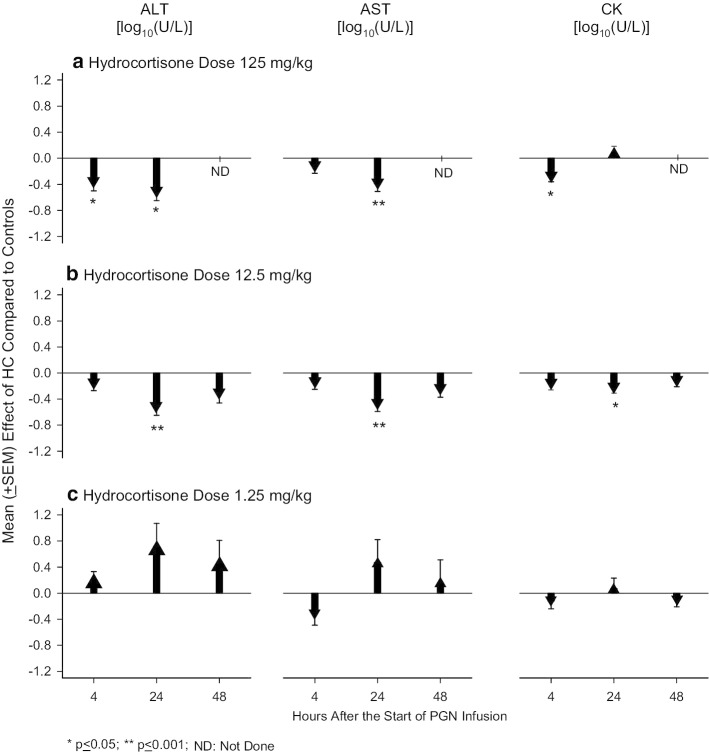
This figure shows the mean effect (± SEM) of either a high (125 mg/kg) (**a**), medium (12.5 mg/kg) (**b**) or low (1.25 mg/kg) (**c**) dose of hydrocortisone (HC) compared to diluent (controls) on blood levels of alanine aminotransferase (ALT), aspartate aminotransferase (AST) and creatine phosphokinase (CK) [all log_10_(pg/ml)] at 4 and 24 h for high-dose hydrocortisone and 4, 24 and 48 h for medium and low doses following the start of a 24-h peptidoglycan infusion. Down and upgoing going arrows indicate the amount that hydrocortisone decreased or increased, respectively, the parameter measured compared to control

Consistent with their effects on intravascular inflammatory markers, high and medium-dose HC inhibited evidence of liver and muscle injury caused by PGN. Compared to controls, high-dose HC significantly decreased alanine aminotransferase (ALT) at 4 and 24 h, aspartate aminotransferase (AST) at 24 h and CK at 4 h (*p* ≤ 0.05) (Fig. 3, Additional file 1: Table S3). Medium-dose HC decreased ALT, AST, and CK at 4, 24 and 48 h and these decreases were significant for each parameter at 24 h (*p* ≤ 0.05) as well as when averaged over the three time points (*p* ≤ 0.01). Low-dose HC did not alter these parameters significantly (*p* > 0.05 for all).

Compared to controls, high and medium but not low-dose HC produced several other significant changes on measured parameters (Table 1 and Additional file 1: Table S4). Consistent with corticosteroid’s hyperglycemic effects, high and medium-dose HC increased blood glucose at 4 or 24 h, respectively (*p* ≤ 0.05). High-dose HC increased total circulating WBC, neutrophils and lymphocytes at 4 h and platelets at 4 and 24 h while medium-dose HC increased neutrophils at 4 h, but decreased lymphocytes and total circulating WBC at 24 h (*p* ≤ 0.05). High-dose HC decreased serum chloride at 4 and 24 h while medium dose increased chloride at 4 h and decreased blood urea nitrogen at 24 h (*p* ≤ 0.05). High-dose HC increased arterial carbon dioxide at 4 and 24 h and bicarbonate at 24 h, while medium-dose HC decreased both measures at 4 h *p* ≤ 0.05).

**Table 1:** Mean (± SEM) effect of high (125 mg/kg), medium (12.5 mg/kg), or low (1.25 mg/kg) hydrocortisone compared to controls on glucose, leukocytes, platelets, creatinine, blood urea nitrogen, electrolytes, and arterial blood gas parameters at 4, 24 and 48 h after the start of peptidoglycan infusion

HC dose (mg/kg)	Time (h)	Glucose [log_10_(mg/dl)]	White blood cells [log_10_(×10^3^/µl)]	Neutrophils [log_10_(×10^3^/µl)]	Lymphocytes [log_10_(×10^3^/µl)]	Platelets [log_10_(×10^3^/µl)]
125	4	0.20 ± 0.07*	0.32 ± 0.05**	0.59 ± 0.08**	0.10 ± 0.05*	0.13 ± 0.06*
	24	0.15 ± 0.08	0.03 ± 0.06	0.10 ± 0.08	0.001 ± 0.05	0.19 ± 0.06*
	48	ND	ND	ND	ND	ND
12.5	4	0.01 ± 0.02	0.06 ± 0.06	0.28 ± 0.07**	− 0.04 ± 0.06	0.09 ± 0.07
	24	0.05 ± 0.02*	− 0.18 ± 0.06*	− 0.14 ± 0.08	− 0.17 ± 0.07*	− 0.07 ± 0.07
	48	0.02 ± 0.03	− 0.01 ± 0.08	0.11 ± 0.10	− 0.05 ± 0.09	0.02 ± 0.09
1.25	4	0.10 ± 0.07	0.03 ± 0.06	− 0.05 ± 0.14	0.08 ± 0.07	− 0.41 ± 0.19
	24	− 0.08 ± 0.08	0.14 ± 0.11	0.10 ± 0.18	0.20 ± 0.11	− 0.37 ± 0.18
	48	− 0.01 ± 0.09	− 0.14 ± 0.11	0.02 ± 0.18	− 0.13 ± 0.12	− 0.41 ± 0.19

		Creatinine (µg/dl)	BUN [log_10_(mg/dl)]	Na (mmol/l)	K (mmol/l)	Cl (mmol/l)
125	4	47.1 ± 37.8	0.07 ± 0.05	− 1.1 ± 1.5	− 0.5 ± 0.3	− 2.6 ± 1.2*
	24	64.1 ± 40.4	− 0.07 ± 0.06	0.2 ± 1.6	− 0.4 ± 0.3	− 4.2 ± 1.4*
	48	ND	ND	ND	ND	ND
12.5	4	− 65 ± 89.1	− 0.02 ± 0.04	0.8 ± 0.5	− 0.1 ± 0.1	1.9 ± 0.6*
	24	50.1 ± 97	− 0.14 ± 0.04*	− 1.0 ± 0.6	0.03 ± 0.11	− 0.1 ± 0.7
	48	ND	− 0.05 ± 0.05	− 0.3 ± 0.7	0.2 ± 0.1	− 0.1 ± 0.9
1.25	4	34.8 ± 66.9	− 0.002 ± 0.09	0.6 ± 1.2	− 0.4 ± 0.4	− 0.34 ± 1.5
	24	141.3 ± 108.8	0.11 ± 0.15	− 0.3 ± 1.9	− 0.2 ± 0.6	1.9 ± 2.4
	48	− 114.2 ± 113.7	0.11 ± 0.15	2.4 ± 2.0	− 0.5 ± 0.6	2.1 ± 2.5

		pH	PCO_2_ (mmHg)	HCO_3_ (mmol/l)	Lac [log_10_(mmol/l)]	PO_2_ (mmHg)
125	4	− 0.02 ± 0.01	2.4 ± 0.9*	0.8 ± 0.7	− 0.001 ± 0.06	− 2.5 ± 5.2
	24	0.001 ± 0.01	2.2 ± 0.9*	2.1 ± 0.7*	− 0.09 ± 0.07	1.5 ± 5.8
	48	ND	ND	ND	ND	ND
12.5	4	− 0.001 ± 0.01	− 1.9 ± 0.6*	− 1.5 ± 0.4*	0.02 ± 0.04	1.5 ± 3.3
	24	0.01 ± 0.01	− 0.7 ± 0.7	0.02 ± 0.4	− 0.05 ± 0.05	− 3.8 ± 3.9
	48	− 0.01 ± 0.01	0.3 ± 0.9	− 0.2 ± 0.6	− 0.001 ± 0.06	0.4 ± 5.1
1.25	4	0.06 ± 0.07	− 1.8 ± 2.3	1.2 ± 1.6	− 0.05 ± 0.16	6.0 ± 12.8
	24	0.01 ± 0.08	1.9 ± 3.7	− 0.2 ± 2.6	0.12 ± 0.22	− 2.0 ± 16.1
	48	0.03 ± 0.08	0.9 ± 3.9	− 0.1 ± 2.7	0.01 ± 0.24	− 2.5 ± 16.5

HC, hydrocortisone; BUN, blood urea nitrogen; Na, sodium; K, potassium; Cl, chloride, PaCO_2_, arterial carbon dioxide; HCO_3_, bicarbonate; PaO_2_, arterial oxygen

* *p* ≤ 0.05; ** *p* ≤ 0.001

### Effects of TNFsr

Following initiation of PGN challenge, compared to diluent controls, survival was increased with each TNFsr dose, but none significantly as follows; high dose [3 survivors of 11 total controls (27.3% survival) vs. 4 of 11 TNFsr 2000 μg/kg animals (36.4% survival) *p* = 0.46); medium dose [5 of 22 controls (22.7% survival) vs. 6 of 22 TNFsr 1000 μg/kg animals (27.3% survival), *p* = 0.26]; and the low dose [ 1 of 9 controls (11.1% survival) vs. 4 of 10 TNFsr 333 μg/kg animals (40% survival) *p* = 0.63] (Additional file 1: Fig. S1). These survival effects were consistent and not significantly different comparing the three TNFsr doses (*p* = 0.98). Therefore, to increase the power to detect an effect related to this selective anti-inflammatory agent, survival and the results from all other parameters were averaged across TNFsr dose for all further analyses.

**Fig. 4:**
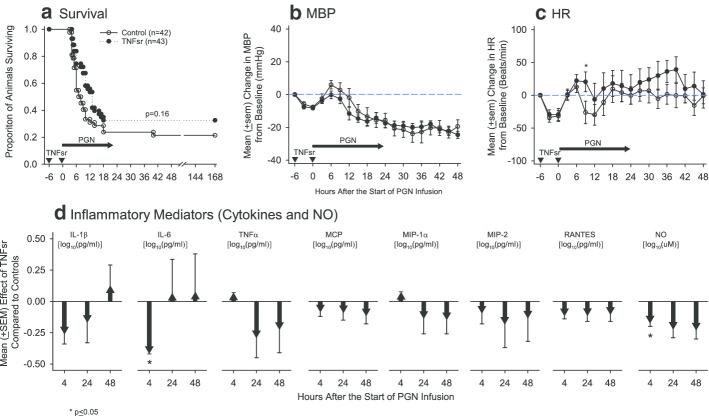
**a**–**c** in this figure compares proportional survival (**a**) and mean (± SEM) serial changes in mean arterial blood pressure (MBP, **b**) and heart rate (HR, **c**) during and after a 24-h *B. anthracis* peptidoglycan infusion (PGN, horizontal arrows) in diluent (control) treated animals versus animals treated with tumor necrosis factor soluble receptor (TNFsr, averaged over the three doses tested; 2000, 1000, and 333 μg/kg). TNFsr or diluent were administered intravenously 6 h before and at the start of PGN infusion (inverted dark arrow heads). Survival is shown from the start of PGN and serial changes in MBP and HR were measured and shown from 6 h before until 48 h after the start of PGN. **d** Shows the mean effect (± SEM) of TNFsr compared to diluent (controls) on blood levels of IL-1β, IL-6, TNFα, MCP, MIP-1α, MIP-2, RANTES [all log_10_(pg/ml)] and nitric oxide [NO, log_10_(μM)] at 4, 24 and 48 h following the start of the 24-h peptidoglycan infusion. Down and upgoing going arrows indicate the amount that TNFsr decreased or increased, respectively, the parameter measured compared to control

Overall, compared to controls, TNFsr did increase survival in a positive trend (*p* = 0.16), but did not alter MBP significantly at any time point (*p* > 0.05 for all) (Fig. 4). However, TNFsr did increase HR from 6 to 45 h after starting PGN and this was significant at 9 h (*p* = 0.04). Notably, TNFsr was associated with decreases in cytokines and NO at most time points measured and decreases in IL-6 and NO were both significant at 4 h (*p* ≤ 0.05, Fig. 4 and Additional file 1: Table S5). TNFsr also decreased [mean (± SEM) effect of TNFsr compared to control] lactate [log_10_(mmol/l)] at both 4 and 48 h (− 0.18 ± 0.06 and − 0.21 ± 0.09) and increased serum sodium at 24 h (1.6 ± 0.6 mmol/l) (*p* ≤ 0.05. Table 2 and Additional file 1: Table S6). Compared to controls, TNFsr did not alter any other parameter measured at any time point significantly (*p* > 0.05 for all).

**Table 2:** Mean (± SEM) effect of TNFsr averaged over the three doses tested compared to controls on alanine and aspartate amino transferases, creatine phosphokinase, glucose, leukocytes, platelets, creatinine, blood urea nitrogen, electrolytes, and arterial blood gas parameters at 4, 24 and 48 h after the start of peptidoglycan infusion

Time (h)	ALT [log_10_(U/l)]	AST [log_10_(U/l)]	CK [log_10_(U/l)]	Glucose [log_10_(mg/dl)]	White blood cells [log_10_(×10^3^/µl)]	Neutrophils [log_10_(×10^3^/µl)]
4	− 0.11 ± 0.12	− 0.10 ± 0.11	− 0.05 ± 0.07	− 0.001 ± 0.04	0.03 ± 0.04	0.15 ± 0.08
24	− 0.05 ± 0.22	0.01 ± 0.21	0.03 ± 0.13	0.06 ± 0.05	0.12 ± 0.08	0.15 ± 0.14
48	0.03 ± 0.23	− 0.10 ± 0.22	− 0.08 ± 0.13	0.05 ± 0.09	− 0.02 ± 0.09	− 0.01 ± 0.16

	Lymphocytes [log_10_ (×10^3^/µl)]	Platelets [log_10_(×10^3^/µl)]	Creatinine (µg/dl)	BUN [log_10_(mg/dl)]	Na (mmol/l)	K (mmol/l)
4	0.02 ± 0.04	0.04 ± 0.07	− 20.4 ± 40.7	− 0.03 ± 0.04	0.5 ± 0.03	− 0.4 ± 0.3
24	0.12 ± 0.07	0.23 ± 0.12	3.8 ± 80.3	0.08 ± 0.07	1.6 ± 0.6*	− 0.3 ± 0.3
48	0.004 ± 0.09	0.20 ± 0.14	35.7 ± 83.1	− 0.04 ± 0.07	0.7 ± 0.6	− 0.4 ± 0.3

	Cl (mmol/l)	pH	PaCO_2_ (mmHg)	HCO_3_ (mmol/L)	Lactate [log(mmol/L)]	PaO_2_ (mmHg)
4	0.2 ± 0.5	0.01 ± 0.02	2.1 ± 1.3	1.7 ± 1.0	− 0.18 ± 0.06*	− 5.6 ± 3.4
24	− 0.1 ± 1.0	0.03 ± 0.04	− 0.4 ± 2.5	1.9 ± 1.7	− 0.03 ± 0.09	− 1.3 ± 6.4
48	1.0 ± 1.0	− 0.02 ± 0.04	1.7 ± 2.6	1.7 ± 1.7	− 0.21 ± 0.09*	2.5 ± 6.6

ALT and AST, alanine and aspartate aminotransferases; CK, creatine phosphokinase; BUN, blood urea nitrogen; Na, sodium; K, potassium; Cl, chloride; PaCO_2_, arterial carbon dioxide; HCO_3_, bicarbonate; PaO_2_, arterial oxygen

* *p* ≤ 0.05

## Discussion

Although an early maladaptive host inflammatory response has been closely associated with the pathogenesis of many lethal bacteria, its role during *B. anthracis* infection is unclear. Lethal and edema toxins appear central to the lethality of *B. anthracis*, but not only elicit little host inflammation, actually inhibit the innate immune response and may promote early infection [7, 29–31]. However, our present findings support the possibility that excessive host inflammation stimulated by *B. anthracis* PGN also contributes to this bacteria’s lethality, an effect that may become stronger as the host’s bacterial cell wall burden increases. High and medium-dose HC limited PGN-associated intravascular inflammatory cytokine and NO levels while improving survival, hemodynamic function and evidence of liver and muscle injury. Administration of TNFsr, a selective anti-inflammatory agent, had limited effects, but was associated with a trend towards increased survival and with early reductions in IL-6 and NO when examined across the three doses tested.

The effectiveness of high and medium-dose HC in the present study likely relates to the direct effect endogenous or exogenous glucocorticoids have on inhibiting nuclear factor-κβ (NF-κβ), a transcription factor central to host cell proinflammatory responses. Peptidoglycan from *B. anthracis* and other Gram-positive bacteria, is a glycan polymer composed of two alternating monomers: N-acetylmuramic acid and *N*-acetylglucosamine [32, 33]. These monomers are joined by short stem peptides (4–5 l- and d-amino acids) that vary by bacteria type. The polymer also includes polysaccharides and lipoteichoic acid, both of which vary by bacteria type. Peptidoglycan and its components, together or individually, represent pathogen-associated molecular patterns (PAMPs) that can elicit innate and adaptive inflammatory responses via their interactions with immune cell membrane or cytosolic pattern-recognition receptors (PRRs) including toll-like receptor-2 (TLR-2), nucleotide-binding oligomerization domains 1 and 2 (NOD-1 and NOD-2), respectively, and cryopyrin [32–35]. These PRRs ultimately all signal through NF-κβ. Activation of NF-κβ upregulates more than 100 genes including ones for all of the inflammatory cytokines and chemokines measured here as well as inducible NO synthase that generates NO [36–39]. Glucocorticoid binding to glucocorticoid receptor-α (GRα) is a primary inhibitor of NF-κβ and its downstream inflammatory effects [40–42].

Consistent with our prior and present studies, administration of *S. aureus* PGN and lipoteichoic acid in a rat model produced lethality (60% mortality), increases in circulating TNFα and NO levels and hepatic injury [43]. Administration of dexamethasone 3 mg/kg (comparable to HC 75 mg/kg in the present model) 2 h before PGN challenge prevented lethality, increased blood pressure and reduced TNFα and NO levels and hepatic injury.

It is possible that high and medium-dose HC had protective effects here besides anti-inflammatory ones. Hydrocortisone has mineralocorticoid effects which could have promoted sodium reabsorption and provided protective increases in intravascular volume. However, hemodynamic changes related to mineralocorticoids typically require several days to develop and sodium and potassium levels were not altered by high or medium-dose HC [44]. Alternatively, HC could have increased vascular smooth muscle responsiveness to endogenous catecholamines [44]. But blood pressure increases with the medium HC dose were not evident until after the start of PGN and its associated inflammatory cytokine and NO production.

We previously showed that 24 h *B. anthracis* PGN infusions similar to the present study produced significant increases in TNFα levels at 6 h that persisted at non-significant levels at 24 and 48 h [12]. While TNFsr had effects on survival, IL-6 and NO in the present study that may implicate TNF in the lethal pathogenesis of *B. anthracis* PGN, this survival effect was far less pronounced than we observed with a lower TNFsr dose in *E. coli* challenged rats treated with antibiotics [28]. Lower TNFsr doses also improved survival significantly in antibiotic-treated mice challenged with *S. aureus *[45]. One interpretation of these findings is that other bacterial components present during a live bacterial challenge elicit lethal responses which are more TNFα dependent than with PGN alone.

Although this study suggests that host inflammatory mediator release is related to the lethal effects of *B. anthracis* PGN, it was not designed to investigate whether anti-inflammatory agents like HC or TNFsr have a therapeutic role. With the present model, such a study would have required anti-inflammatory agents be started no earlier than the start of the PGN infusion, and ideally sometime following that to simulate a patient with progressive infection. Furthermore, the potential adverse inhibitory effects of these agents on host defense and microbial clearance could only be tested in a model of live bacterial infection that would ideally also include antibiotic and other standard therapies. However, the present findings do have clinical implications. Patients progressing to severe anthrax infection have extensive extravascular fluid accumulation manifested as pleural and peritoneal effusions and tissue edema [4, 17, 46, 47]. Even though evidence suggests that lethal toxin disrupts endothelial barrier function, PGN-stimulated intravascular inflammation and coagulopathy could also contribute to this endothelial dysfunction [46, 48, 49]. Such inflammation is known to disrupt endothelial gap junction function and allows extravasation of protein and fluid [50, 51]. Consistent with that, *B. anthracis* PGN administration in baboons was shown to disrupt endothelial gap junctions and increase vascular permeability measured with fluorescent labeled albumin [15]. At this time, based only on several small observational clinical studies, the CDC recommends consideration of corticosteroid use in patients with head and neck *B. anthracis* infection producing compromising edema of the airway [52]. The present findings are the first preclinical evidence we are aware of to provide support for this CDC recommendation.

In this and our previous present study, a highly lethal PGN dose infused over 24 h produced an early increase in circulating inflammatory cytokines that gradually subsided over 24 to 48 h. Under these conditions, HC and TNFsr had anti-inflammatory effects with beneficial effects on survival and organ injury. However, PGN, like lipopolysaccharide, under come conditions, has been shown to have immunosuppressive effects and may actually augment host defenses [53, 54]. Treatment strategies targeting PGN with *B. anthracis* or other Gram-positive bacteria, must account for the conditions under which this cell wall component elicits maladaptive harmful as opposed to adaptive beneficial host responses.

This study has limitations. First, it is unknown how the concentration of PGN employed in the present study relates to the levels occurring during live *B. anthracis* infection. The doses we employed in this and our prior study are comparable though to doses of *B. anthracis* PGN employed in a baboon model [12, 15]. Challenge with PGN in that model simulated changes associated with live *B. anthracis* challenge in a similar non-human primate model [7]. Second, the PGN preparation used here had very small amounts of LPS contamination. However, an LPS concentration greater than the highest level in the PGN batches employed produced no lethality and no significant changes in any of the same inflammatory markers measured in the HC and TNFsr experiments except for one. By contrast, the dose of PGN employed produced highly significant changes in all of these measures. Third, limitations on animal numbers and purified PGN prevented formal power analysis calculations for experiments as well as testing whether larger sample sizes or a wider range of treatment doses would provide stronger evidence regarding the effects of TNFsr or the lowest HC dose investigated. These restrictions also prevented determining whether HC or TNFs had inhibitory effects on PGN-associated coagulopathy, tests which would have required additional PGN and animals for sampling. This limitation also prevented further exploration of a basis for the possible trend in survival and its associated inflammatory response noted with the lowest TNFsr dose studied. Fourth, blood measures with high-dose HC were only obtained over 24 h and the treatment’s effects on inflammatory mediators at 48 h are unclear. Finally, this model tested PGN alone, and as stated above, both LT and ET can inhibit components in the innate immune response which could alter the effectiveness of anti-inflammatory therapies themselves.

## Conclusion

Invasive *B. anthracis* infection is associated with resistant shock and high lethality rates. While evidence supports the role LT and ET have in this lethality*,* these toxins do not produce the excessive intravascular inflammatory response observed in animals challenged with lethal *B. anthracis* doses. The present findings support the possibility that inflammation caused by *B. anthracis* cell wall PGN has lethal effects. The high bacterial loads patients and animals dying with *B. anthracis* provide a large PGN source. Further studies are necessary to determine whether anti-inflammatory agents like HC or more selective ones, add to the protective effects of standard and toxin directed therapies during *B. anthracis* infection itself.

## Disclaimer

The opinions expressed in this article are those of the authors and do not represent any position or policy of the National Institutes of Health, the US Department of Health and Human Services, or the US government.

## Supplementary information

**Additional file 1. **Additional tables and figure.

## Data Availability

The datasets used and/or analyzed during the current study are available from the corresponding author on reasonable request.

## Abbreviations

PGN: Peptidoglycan
IV: Intravenous
HC: Hydrocortisone
TNFsr: Tumor necrosis factor soluble receptor
LT: Lethal toxin
ET: Edema toxin
MBP: Mean arterial blood pressure
HR: Heart rate
CBC: Complete blood count
ABG: Arterial blood gas
ALT: Alanine aminotransferase
AST: Aspartate aminotransferase
CK: Creatine phosphokinase
BUN: Blood urea nitrogen
HF: Hydrofluoric acid
LPS: Lipopolysaccharide
IL-1β: Interleukin-1β
IL-6: Interleukin-6
TNFα: Tumor necrosis factor α
MCP: Monocyte chemoattractant protein
MIP-1α and MIP-2: Macrophage inflammatory protein-1α and 2
RANTES: Regulated on activation, normal T-cell expressed and secreted
NO: Nitric oxide

## References

[1] Adalja AA, Toner E, Inglesby TV (2015). Clinical management of potential bioterrorism-related conditions. N Engl J Med.

[2] Cui X, Nolen LD, Sun J, Booth M, Donaldson L, Quinn CP, Boyer AE, Hendricks K, Shadomy S, Bothma P, Judd O, McConnell P, Bower WA, Eichacker PQ (2017). Analysis of anthrax immune globulin intravenous with antimicrobial treatment in injection drug users, Scotland, 2009–2010. Emerg Infect Dis.

[3] Sweeney DA, Hicks CW, Cui X, Li Y, Eichacker PQ (2011). Anthrax infection. Am J Respir Crit Care Med.

[4] Booth M, Donaldson L, Cui X, Sun J, Cole S, Dailsey S, Hart A, Johns N, McConnell P, McLennan T, Parcell B, Robb H, Shippey B, Sim M, Wallis C, Eichacker PQ (2014). Confirmed *Bacillus anthracis* infection among persons who inject drugs, Scotland, 2009–2010. Emerg Infect Dis.

[5] Remy KE, Qiu P, Li Y, Cui X, Eichacker PQ (2013). *B. anthracis* associated cardiovascular dysfunction and shock: the potential contribution of both non-toxin and toxin components. BMC Med.

[6] Sweeney DA, Cui X, Solomon SB, Vitberg DA, Migone TS, Scher D, Danner RL, Natanson C, Subramanian GM, Eichacker PQ (2010). Anthrax lethal and edema toxins produce different patterns of cardiovascular and renal dysfunction and synergistically decrease survival in canines. J Infect Dis.

[7] Cui X, Moayeri M, Li Y, Li X, Haley M, Fitz Y, Correa-Araujo R, Banks SM, Leppla SH, Eichacker PQ (2004). Lethality during continuous anthrax lethal toxin infusion is associated with circulatory shock but not inflammatory cytokine or nitric oxide release in rats. Am J Physiol Regul Integr Comp Physiol.

[8] Cui X, Li Y, Li X, Laird MW, Subramanian M, Moayeri M, Leppla SH, Fitz Y, Su J, Sherer K, Eichacker PQ (2007). Bacillus anthracis edema and lethal toxin have different hemodynamic effects but function together to worsen shock and outcome in a rat model. J Infect Dis.

[9] Coggeshall KM, Lupu F, Ballard J, Metcalf JP, James JA, Farris D, Kurosawa S (2013). The sepsis model: an emerging hypothesis for the lethality of inhalation anthrax. J Cell Mol Med.

[10] Stearns-Kurosawa DJ, Lupu F, Taylor FB, Kinasewitz G, Kurosawa S (2006). Sepsis and pathophysiology of anthrax in a nonhuman primate model. Am J Pathol.

[11] Cui X, Su J, Li Y, Shiloach J, Solomon S, Kaufman JB, Mani H, Fitz Y, Weng J, Altaweel L, Besch V, Eichacker PQ (2010). Bacillus anthracis cell wall produces injurious inflammation but paradoxically decreases the lethality of anthrax lethal toxin in a rat model. Intensive Care Med.

[12] Qiu P, Li Y, Shiloach J, Cui X, Sun J, Trinh L, Kubler-Kielb J, Vinogradov E, Mani H, Al-Hamad M, Fitz Y, Eichacker PQ (2013). *Bacillus anthracis* Cell wall peptidoglycan but not lethal or edema toxins produces changes consistent with disseminated intravascular coagulation in a rat model. J Infect Dis.

[13] Iyer JK, Coggeshall KM (2011). Cutting edge: primary innate immune cells respond efficiently to polymeric peptidoglycan, but not to peptidoglycan monomers. J Immunol.

[14] Langer M, Malykhin A, Maeda K, Chakrabarty K, Williamson KS, Feasley CL, West CM, Metcalf JP, Coggeshall KM (2008). *Bacillus anthracis* peptidoglycan stimulates an inflammatory response in monocytes through the p38 mitogen-activated protein kinase pathway. PLoS ONE.

[15] Popescu NI, Silasi R, Keshari RS, Girton A, Burgett T, Zeerleder SS, Gailani D, Gruber A, Lupu F, Coggeshall KM (2018). Peptidoglycan induces disseminated intravascular coagulation in baboons through activation of both coagulation pathways. Blood.

[16] Iyer JK, Khurana T, Langer M, West CM, Ballard JD, Metcalf JP, Merkel TJ, Coggeshall KM (2010). Inflammatory cytokine response to *Bacillus anthracis* peptidoglycan requires phagocytosis and lysosomal trafficking. Infect Immun.

[17] Abramova FA, Grinberg LM, Yampolskaya OV, Walker DH (1993). Pathology of inhalational anthrax in 42 cases from the Sverdlovsk outbreak of 1979. Proc Natl Acad Sci USA.

[18] Grinberg LM, Abramova FA, Yampolskaya OV, Walker DH, Smith JH (2001). Quantitative pathology of inhalational anthrax I: quantitative microscopic findings. Mod Pathol.

[19] Twenhafel NA (2010). Pathology of inhalational anthrax animal models. Vet Pathol.

[20] Ekwunife FS, Singh J, Taylor KG, Doyle RJ (1991). Isolation and purification of cell wall polysaccharide of *Bacillus anthracis* (delta Sterne). FEMS Microbiol Lett.

[21] Nair AB, Jacob S (2016). A simple practice guide for dose conversion between animals and human. J Basic Clin Pharm.

[22] Briegel J, Forst H, Haller M, Schelling G, Kilger E, Kuprat G, Hemmer B, Hummel T, Lenhart A, Heyduck M, Stoll C, Peter K (1999). Stress doses of hydrocortisone reverse hyperdynamic septic shock: a prospective, randomized, double-blind, single-center study. Crit Care Med.

[23] Minneci PC, Deans KJ, Banks SM, Eichacker PQ, Natanson C (2004). Meta-analysis: the effect of steroids on survival and shock during sepsis depends on the dose. Ann Intern Med.

[24] Petersons CJ, Mangelsdorf BL, Thompson CH, Burt MG (2014). Acute effect of increasing glucocorticoid replacement dose on cardiovascular risk and insulin sensitivity in patients with adrenocorticotrophin deficiency. J Clin Endocrinol Metab.

[25] van den Brink HR, van Wijk MJ, Geertzen RG, Bijlsma JW (1994). Influence of corticosteroid pulse therapy on the serum levels of soluble interleukin 2 receptor, interleukin 6 and interleukin 8 in patients with rheumatoid arthritis. J Rheumatol.

[26] Lequerre T, Farran E, Menard JF, Kozyreff-Meurice M, Vandhuick T, Tharasse C, Pouplin S, Daragon A, Le Loet X, Varin R, Vittecoq O (2015). Switching from an anti-TNF monoclonal antibody to soluble TNF-receptor yields better results than vice versa: an observational retrospective study of 72 rheumatoid arthritis switchers. Joint Bone Spine.

[27] Lim MJ, Kwon SR, Joo K, Son MJ, Park SG, Park W (2014). Early effects of tumor necrosis factor inhibition on bone homeostasis after soluble tumor necrosis factor receptor use. Korean J Intern Med.

[28] Qiu P, Li Y, Ding Y, Weng J, Banks SM, Kern S, Fitz Y, Suffredini AF, Eichacker PQ, Cui X (2011). The individual survival benefits of tumor necrosis factor soluble receptor and fluid administration are not additive in a rat sepsis model. Intensive Care Med.

[29] Cui X, Li Y, Li X, Haley M, Moayeri M, Fitz Y, Leppla SH, Eichacker PQ (2006). Sublethal doses of *Bacillus anthracis* lethal toxin inhibit inflammation with lipopolysaccharide and *Escherichia coli* challenge but have opposite effects on survival. J Infect Dis.

[30] Erwin JL, DaSilva LM, Bavari S, Little SF, Friedlander AM, Chanh TC (2001). Macrophage-derived cell lines do not express proinflammatory cytokines after exposure to *Bacillus anthracis* lethal toxin. Infect Immun.

[31] Pellizzari R, Guidi-Rontani C, Vitale G, Mock M, Montecucco C (1999). Anthrax lethal factor cleaves MKK3 in macrophages and inhibits the LPS/IFNgamma-induced release of NO and TNFalpha. FEBS Lett.

[32] McDonald C, Inohara N, Nunez G (2005). Peptidoglycan signaling in innate immunity and inflammatory disease. J Biol Chem.

[33] Weidenmaier C, Peschel A (2008). Teichoic acids and related cell-wall glycopolymers in Gram-positive physiology and host interactions. Nat Rev Microbiol.

[34] Franchi L, Park JH, Shaw MH, Marina-Garcia N, Chen G, Kim YG, Nunez G (2008). Intracellular NOD-like receptors in innate immunity, infection and disease. Cell Microbiol.

[35] Loving CL, Osorio M, Kim YG, Nunez G, Hughes MA, Merkel TJ (2009). Nod1/Nod2-mediated recognition plays a critical role in induction of adaptive immunity to anthrax after aerosol exposure. Infect Immun.

[36] Baeuerle PA, Baltimore D (1988). Activation of DNA-binding activity in an apparently cytoplasmic precursor of the NF-kappa B transcription factor. Cell.

[37] Fan J, Ye RD, Malik AB (2001). Transcriptional mechanisms of acute lung injury. Am J Physiol Lung Cell Mol Physiol.

[38] Liu SF, Malik AB (2006). NF-kappa B activation as a pathological mechanism of septic shock and inflammation. Am J Physiol Lung Cell Mol Physiol.

[39] Meduri GU, Annane D, Chrousos GP, Marik PE, Sinclair SE (2009). Activation and regulation of systemic inflammation in ARDS: rationale for prolonged glucocorticoid therapy. Chest.

[40] Ehrchen J, Steinmuller L, Barczyk K, Tenbrock K, Nacken W, Eisenacher M, Nordhues U, Sorg C, Sunderkotter C, Roth J (2007). Glucocorticoids induce differentiation of a specifically activated, anti-inflammatory subtype of human monocytes. Blood.

[41] Galon J, Franchimont D, Hiroi N, Frey G, Boettner A, Ehrhart-Bornstein M, O'Shea JJ, Chrousos GP, Bornstein SR (2002). Gene profiling reveals unknown enhancing and suppressive actions of glucocorticoids on immune cells. FASEB J.

[42] Rhen T, Cidlowski JA (2005). Antiinflammatory action of glucocorticoids–new mechanisms for old drugs. N Engl J Med.

[43] Kengatharan KM, De Kimpe SJ, Thiemermann C (1996). Role of nitric oxide in the circulatory failure and organ injury in a rodent model of gram-positive shock. Br J Pharmacol.

[44] Pirpiris M, Sudhir K, Yeung S, Jennings G, Whitworth JA (1992). Pressor responsiveness in corticosteroid-induced hypertension in humans. Hypertension.

[45] Fei Y, Wang W, Kwiecinski J, Josefsson E, Pullerits R, Jonsson IM, Magnusson M, Jin T (2011). The combination of a tumor necrosis factor inhibitor and antibiotic alleviates staphylococcal arthritis and sepsis in mice. J Infect Dis.

[46] Cui X, Xu W, Neupane P, Weiser-Schlesinger A, Weng R, Pockros B, Li Y, Moayeri M, Leppla SH, Fitz Y, Eichacker PQ (2019). Bacillus anthracis lethal toxin, but not edema toxin, increases pulmonary artery pressure and permeability in isolated perfused rat lungs. Am J Physiol Heart Circ Physiol.

[47] Jernigan JA, Stephens DS, Ashford DA, Omenaca C, Topiel MS, Galbraith M, Tapper M, Fisk TL, Zaki S, Popovic T, Meyer RF, Quinn CP, Harper SA, Fridkin SK, Sejvar JJ, Shepard CW, McConnell M, Guarner J, Shieh WJ, Malecki JM, Gerberding JL, Hughes JM, Perkins BA, Anthrax Bioterrorism Investigation T (2001). Bioterrorism-related inhalational anthrax: the first 10 cases reported in the United States. Emerg Infect Dis.

[48] Suffredini DA, Cui X, Xu W, Li Y, Eichacker PQ (2017). The potential pathogenic contributions of endothelial barrier and arterial contractile dysfunction to shock due to *B. anthracis* lethal and edema toxins. Toxins..

[49] Tian Y, Mambetsariev I, Sarich N, Meng F, Birukova AA (2015). Role of microtubules in attenuation of PepG-induced vascular endothelial dysfunction by atrial natriuretic peptide. Biochim Biophys Acta.

[50] Kumar P, Shen Q, Pivetti CD, Lee ES, Wu MH, Yuan SY (2009). Molecular mechanisms of endothelial hyperpermeability: implications in inflammation. Expert Rev Mol Med.

[51] Schnoor M, Garcia Ponce A, Vadillo E, Pelayo R, Rossaint J, Zarbock A (2017). Actin dynamics in the regulation of endothelial barrier functions and neutrophil recruitment during endotoxemia and sepsis. Cell Mol Life Sci.

[52] Hendricks KA, Wright ME, Shadomy SV, Bradley JS, Morrow MG, Pavia AT, Rubinstein E, Holty JE, Messonnier NE, Smith TL, Pesik N, Treadwell TA, Bower WA, Workgroup on Anthrax Clinical G (2014). Centers for disease control and prevention expert panel meetings on prevention and treatment of anthrax in adults. Emerg Infect Dis..

[53] Nakayama K, Okugawa S, Yanagimoto S, Kitazawa T, Tsukada K, Kawada M, Kimura S, Hirai K, Takagaki Y, Ota Y (2004). Involvement of IRAK-M in peptidoglycan-induced tolerance in macrophages. J Biol Chem.

[54] Murphey ED, Sherwood ER (2008). Pretreatment with the Gram-positive bacterial cell wall molecule peptidoglycan improves bacterial clearance and decreases inflammation and mortality in mice challenged with Pseudomonas aeruginosa. Microbes Infect.

