# Supplementary material for: Hydrocortisone decreases lethality and inflammatory cytokine and nitric oxide production in rats challenged with B. anthracis cell wall peptidoglycan
Source: Intensive Care Med Exp. 2020 Nov 18;8:67. doi: 10.1186/s40635-020-00358-4 (PMC7674536; doi:10.1186/s40635-020-00358-4)
Supplement: Supplementary file 1 — Additional file 1. Additional tables and figure. [file 40635_2020_358_MOESM1_ESM.pdf]

Supplemental Figure 1

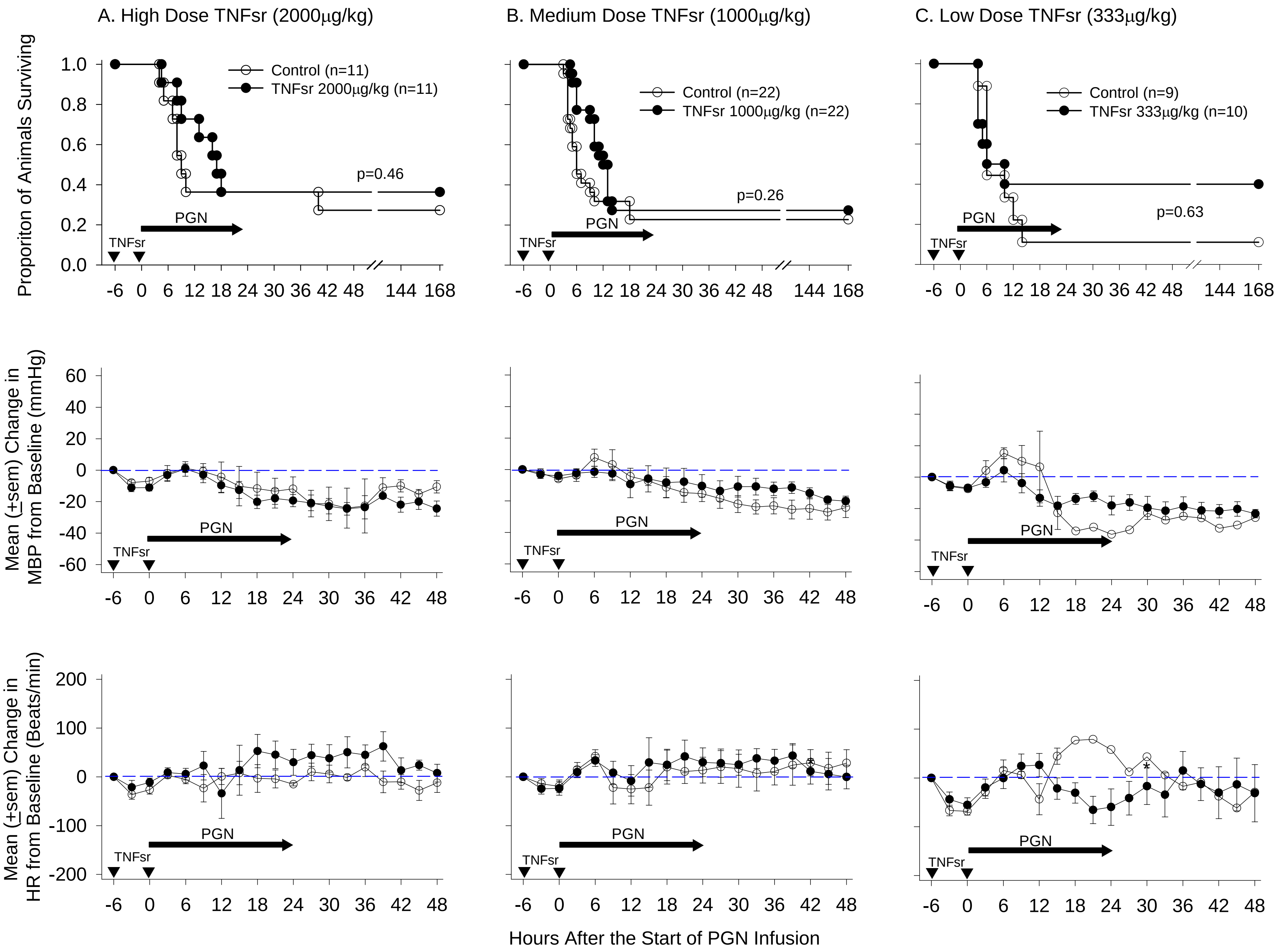

Supplemental Table 1. Summary of Numbers of Animals Studied

Hydrocortisone					
		Numbers of Animals			
Experiment	Dose (mg/kg)	Treatment		Control	
		Total	Survivor	Total	Survivor
1	High (125)	6	6	5	3
2	High (125)	6	6	6	4
3	High (125)	5	5	6	4
4	High (125)	5	5	6	4
5	Medium (12.5)	5	5	4	3
6	Medium (12.5)	6	6	5	0
7	Medium (12.5)	5	5	5	2
8	Medium (12.5)	5	5	5	5
9	Medium (12.5)	4	4	4	1
10	Low (1.25)	6	1	4	1
11	Low (1.25)	5	4	5	2

TNFsr					
Experiment	Dose(μg/kg)	Treatment		Control	
		Total	Survivor	Total	Survivor
1	High (2000)	5	1	6	2
2	High (2000)	6	3	5	1
3	Medium (1000)	5	3	5	0
4	Medium (1000)	5	1	6	1
5	Medium (1000)	6	1	6	3
6	Medium (1000)	6	1	5	1
7	Low (333)	4	2	4	0
8	Low (333)	6	2	5	1

Supplemental Table 2. Summary of mean [$\log_{10}$(pg/ml)±SEM] cytokine and nitric oxide levels comparing control and high, medium or low dose hydrocortisone (HC) groups at 4, 24 and 48h after the start of peptidoglycan infusion

		Control	HC	Control	HC	Control	HC	Control	HC
HC Dose (mg/kg)	Time (h)	IL-1β		IL-6		TNFα		MCP-1	
125	4	2.47±0.13	1.71±0.06	1.35±0.21	0.34±0.13	1.55±0.15	0.92±0.10	4.27±0.05	3.83±0.06
	24	1.43±0.15	1.27±0.12	0.48±0.01	0.48±0.01	0.67±0.19	0.71±0.09	3.72±0.10	3.81±0.04
	48	ND	ND	ND	ND	ND	ND	ND	ND
		MIP-1α		MIP-2		RANTES		NO	
	4	2.93±0.04	2.68±0.09	2.03±0.15	1.04±0.11	3.54±0.05	3.13±0.03	1.78±0.05	1.64±0.07
	24	2.33±0.17	1.89±0.16	0.50±0.25	0.28±0.12	2.93±0.05	2.92±0.03	1.67±0.07	1.55±0.03
	48	ND	ND	ND	ND	ND	ND	ND	ND
12.5		IL-1β		IL-6		TNFα		MCP-1	
	4	2.30±0.15	2.11±0.12	0.99±0.18	0.30±0.11	1.82±0.15	1.71±0.17	4.07±0.05	4.03±0.03
	24	1.48±0.16	1.50±0.16	0.29±0.06	0.34±0.02	0.91±0.23	1.19±0.18	3.69±0.07	3.71±0.04
	48	1.30±0.24	1.22±0.23	0.27±0.04	0.30±0.01	1.09±0.26	1.02±0.29	3.30±0.05	3.32±0.05
		MIP-1α		MIP-2		RANTES		NO	
	4	2.77±0.10	2.81±0.03	1.67±0.17	1.51±0.09	3.45±0.05	3.32±0.05	1.32±0.08	1.01±0.11
	24	2.30±0.12	2.40±0.09	0.42±0.23	0.53±0.15	2.99±0.07	3.01±0.06	1.56±0.09	1.50±0.06
	48	1.66±0.12	1.63±0.10	-0.02±0.19	0.10±0.20	3.05±0.08	2.88±0.08	1.18±0.23	1.34±0.14
1.25		IL-1β		IL-6		TNFα		MCP-1	
	4	2.65±0.23	2.54±0.08	1.73±0.42	1.56±0.31	1.93±0.22	2.05±0.13	4.04±0.08	4.05±0.05
	24	1.34±0.44	1.85±0.29	0.33±0.01	0.32±0.01	0.74±0.38	1.19±0.30	3.53±0.18	3.68±0.13
	48	1.16±0.28	2.05±0.41	0.33±0.01	0.33±0.01	0.64±0.26	1.42±0.43	3.07±0.10	3.29±0.18
		MIP-1α		MIP-2		RANTES		NO	
	4	2.91±0.06	2.86±0.05	2.04±0.19	2.12±0.14	3.36±0.09	3.38±0.07	1.37±0.07	1.24±0.04
	24	2.04±0.58	2.26±0.22	0.71±0.12	0.88±0.25	2.62±0.17	2.84±0.11	1.40±0.11	1.48±0.23
	48	1.37±0.16	1.64±0.17	-0.11±0.22	0.65±0.34	2.57±0.03	2.84±0.06	1.31±0.06	1.33±0.35

IL-1β - interleukin-1β; IL-6 – interleukin-6; TNFα - tumor necrosis factor α; MCP – monocyte chemoattractant protein; MIP-1α and MIP-2 – macrophage inflammatory protein-1α and 2; RANTES – regulated on activation, normal T-cell expressed and secreted; NO – nitric oxide

Supplemental Table 3. Summary of mean (±SEM) liver function measures comparing control and high, medium or low dose hydrocortisone (HC) groups at 4, 24 and 48h after the start of peptidoglycan infusion

HC Dose (mg/kg)	Time (h)	ALT [log_{10}(U/L)]		AST [log_{10}(U/L)]		CK [log_{10}(U/L)]	
		Control	HC	Control	HC	Control	HC
125	4	1.79±0.14	1.43±0.05	2.17±0.10	1.97±0.05	2.20±0.08	1.93±0.04
	24	1.69±0.13	1.19±0.04	1.98±0.10	1.67±0.07	1.77±0.04	1.76±0.06
	48	ND	ND	ND	ND	ND	ND
12.5	4	1.62±0.06	1.45±0.03	2.10±0.06	1.95±0.05	2.05±0.07	1.94±0.03
	24	1.83±0.22	1.34±0.09	2.28±0.20	1.84±0.08	1.92±0.10	1.81±0.02
	48	1.37±0.10	1.12±0.09	1.77±0.08	1.55±0.05	1.89±0.03	1.81±0.03
1.25	4	1.77±0.18	1.86±0.17	2.45±0.20	2.19±0.10	2.16±0.11	2.04±0.06
	24	1.14±0.03	1.85±0.31	1.79±0.10	2.37±0.31	1.78±0.05	1.96±0.12
	48	1.14±0.03	1.61±0.29	1.72±0.04	2.01±0.34	1.88±0.04	1.96±0.07

ALT – alanine aminotransferase; AST – aspartate aminotransferase; CK – creatine phosphokinase

Supplemental Table 4. Summary of mean (±SEM) electrolytes, glucose, and arterial blood gas and complete blood count parameters comparing control and high, medium or low dose hydrocortisone (HC) groups at 4, 24 and 48h after the start of peptidoglycan infusion

		Control	HC	Control	HC	Control	HC	Control	HC	Control	HC
HC Dose (mg/kg)	Time (h)	Glucose [log_{10}(mg/dl)]		White Blood Cells [log_{10} (x10^3/μl)]		Neutrophils [log_{10}(x10^3/μl)]		Lymphocytes [log_{10}(x10^3/μl)]		Platelets [log_{10}(x10^3/μl)]	
125	4	1.96±0.07	2.17±0.07	0.48±0.05	0.80±0.03	-0.07±0.06	0.52±0.04	0.29±0.04	0.40±0.04	2.68±0.05	2.83±0.04
	24	2.02±0.06	2.20±0.07	0.74±0.04	0.75±0.03	0.25±0.07	0.29±0.04	0.52±0.04	0.53±0.04	2.37±0.11	2.62±0.08
	48	ND	ND	ND	ND	ND	ND	ND	ND	ND	ND
12.5	4	1.99±0.01	1.99±0.01	0.53±0.06	0.59±0.03	-0.12±0.09	0.15±0.04	0.38±0.06	0.33±0.04	2.51±0.05	2.60±0.04
	24	2.00±0.03	2.04±0.01	0.98±0.06	0.76±0.03	0.51±0.06	0.26±0.03	0.76±0.07	0.58±0.04	2.29±0.09	2.15±0.06
	48	2.02±0.01	2.03±0.02	1.11±0.06	1.04±0.04	0.71±0.05	0.68±0.03	0.84±0.07	0.75±0.06	2.50±0.06	2.41±0.03
1.25	4	1.94±0.04	2.04±0.01	0.07±0.05	0.10±0.04	-0.55±0.13	-0.60±0.07	-0.12±0.04	-0.04±0.05	2.28±0.09	2.18±0.07
	24	2.03±0.01	1.94±0.11	0.74±0.05	0.86±0.08	0.15±0.16	0.11±0.07	0.53±0.03	0.74±0.09	1.95±0.28	1.53±0.11
	48	2.05±0.02	2.02±0.05	1.07±0.07	0.90±0.11	0.55±0.13	0.38±0.13	0.77±0.03	0.64±0.10	2.51±0.22	2.03±0.16
		Creatinine [log_{10}(μg/dl)]		BUN [log_{10}(mg/dl)]		Na (mmol/L)		K (mmol/L)		Cl (mmol/L)	
125	4	2.48±0.04	2.57±0.02	1.10±0.02	1.16±0.03	138±1	137±2	3.5±0.1	2.9±0.1	112±1	109±1
	24	2.45±0.05	2.54±0.04	1.18±0.05	1.12±0.05	138±1	138±2	3.2±0.1	3.3±0.1	114±1	109±1
	48	ND	ND	ND	ND	ND	ND	ND	ND	ND	ND
12.5	4	2.71±0.07	2.71±0.05	0.99±0.03	0.97±0.02	139±1	140±0.2	3.1±0.1	3.0±0.04	112±0.4	114±1
	24	2.74±0.05	2.77±0.02	1.23±0.06	1.10±0.02	141±1	140±0.3	3.3±0.1	3.3±0.1	114±1	114±0.4
	48	2.84±0.02	2.82±0.01	1.15±0.04	1.10±0.05	141±1	141±0.4	3.2±0.1	3.4±0.1	116±1	116±1
1.25	4	2.44±0.08	2.50±0.07	1.04±0.04	1.04±0.03	138±1	139±1	3.8±o.5	3.4±0.2	114±1	113±1
	24	2.45±0.14	2.63±0.08	1.17±0.05	1.29±0.14	140±1	139±1	3.7±0.1	3.4±0.1	113±1	115±1
	48	2.65±0.07	2.50±0.12	1.22±0.05	1.35±0.24	139±1	141±3	4.2±0.1	3.7±0.2	113±1	115±4
		pH		PCO_2 (mmHg)		HCO_3 (mmol/L)		Lactate [log_{10}(mmol/L)]		PO_2(mmHg)	
125	4	7.49±0.01	7.46±0.00	24.2±0.8	26.6±0.4	18.5±0.7	19.3±0.2	0.10±0.06	0.10±0.03	105.7±4.5	103.4±2.1
	24	7.47±0.01	7.48±0.01	25.5±0.7	26.9±0.4	18.9±0.4	20.2±0.4	0.07±0.05	0.01±0.03	109.6±4.5	111.8±4.4
	48	ND	ND	ND	ND	ND	ND	ND	ND	ND	ND
12.5	4	7.48±0.01	7.48±0.00	26.2±0.4	24.3±0.4	19.8±0.3	18.3±0.3	0.10±0.03	0.11±0.02	101.6±3.5	103.2±1.8
	24	7.47±0.01	7.48±0.00	25.8±0.6	25.2±0.3	19.0±0.4	19.0±0.2	0.14±0.06	0.08±0.03	106.4±3.7	102.9±2.0
	48	7.46±0.01	7.48±0.01	26.8±0.6	27.1±0.8	19.2±0.4	19.0±0.4	0.02±0.04	0.02±0.03	104.8±2.7	105.1±2.3
1.25	4	7.43±0.06	7.49±0.04	25.5±2.2	23.6±1.5	17.1±1.6	18.2±1.0	0.25±0.13	0.20±0.11	103.3±13.2	97.3±5.4
	24	7.52±0.00	7.48±0.02	22.4±1.6	24.3±2.0	18.5±1.4	18.3±1.3	-0.06±0.12	0.14±0.09	94.0±2.3	98.4±6.7
	48	7.49±0.00	7.48±0.01	27.0±1.2	27.9±2.1	21.0±0.9	21.0±1.5	-0.09±0.08	-0.03±0.18	104.3±3.8	106.9±7.2

Na – serum sodium; K – serum potassium; Cl – serum chloride; pH – arterial pH; PCO_2 – arterial carbon dioxide; HCO_3 – bicarbonate

Supplemental Table 5. Summary of mean [log_{10}(pg/ml)]±SEM) cytokine and nitric oxide levels comparing control and averaged three doses of TNFsr at 4, 24 and 48h after the start of peptidoglycan infusion

	Control	TNFsr	Control	TNFsr	Control	TNFsr	Control	TNFsr
Time (h)	IL-1β		IL-6		TNFα		MCP-1	
4	3.12±0.10	2.92±0.07	2.26±0.15	1.89±0.15	2.52±0.11	2.53±0.05	4.12±0.05	4.08±0.03
24	2.24±0.22	2.25±0.16	0.33±0.01	0.33±0.01	1.60±0.22	1.62±0.17	3.71±0.10	3.71±0.07
48	1.98±0.34	2.26±0.18	0.32±0.01	0.33±0.01	1.48±0.30	1.63±0.20	3.24±0.11	3.22±0.07
	MIP-1α		MIP-2		RANTES		NO	
4	2.87±0.08	2.88±0.05	2.20±0.11	2.15±0.09	3.59±0.04	3.50±0.03	1.41±0.03	1.27±0.03
24	2.33±0.25	2.32±0.15	0.91±0.07	0.84±0.11	2.99±0.08	2.96±0.05	1.84±0.16	1.67±0.12
48	1.52±0.17	1.60±0.08	0.51±0.24	0.53±0.14	2.93±0.09	2.91±0.05	1.49±0.15	1.30±0.10

IL-1β - interleukin-1β; IL-6 – interleukin-6; TNFα - tumor necrosis factor α; MCP – monocyte chemoattractant protein; MIP-1α and MIP-2 – macrophage inflammatory protein-1α and 2; RANTES – regulated on activation, normal T-cell expressed and secreted; NO – nitric oxide

Supplemental Table 6. Summary of mean (±SEM) electrolytes, glucose, and arterial blood gas and complete blood count parameters comparing control and averaged three doses of TNFsr at 4, 24 and 48h after the start of peptidoglycan infusion

	Control	TNFsr	Control	TNFsr	Control	TNFsr
Time (h)	ALT [$\log_{10}$(U/L)]		AST [$\log_{10}$(U/L)]		CK [$\log_{10}$(U/L)]	
4	1.96±0.09	1.87±0.08	2.31±0.08	2.22±0.08	2.25±0.05	2.21±0.05
24	2.10±0.26	2.06±0.17	2.45±0.25	2.41±0.16	1.98±0.08	2.03±0.11
48	1.62±0.19	1.62±0.09	2.07±0.21	1.89±0.09	2.07±0.11	1.96±0.04

	Control	TNFsr	Control	TNFsr	Control	TNFsr	Control	TNFsr	Control	TNFsr
Time (h)	Glucose [$\log_{10}$(mg/dl)]		WBC [$\log_{10}$(x10^3/µl)]		Neutrophils [$\log_{10}$(x10^3/µl)]		Lymphocytes [$\log_{10}$(x10^3/µl)]		Platelets [$\log_{10}$(x10^3/µl)]	
4	1.97±0.03	1.97±0.02	0.19±0.03	0.23±0.03	-0.65±0.07	-0.50±0.05	0.08±0.02	0.09±0.03	2.16±0.04	2.20±0.05
24	1.92±0.05	1.98±0.03	0.98±0.06	1.11±0.05	0.35±0.09	0.45±0.06	0.82±0.07	0.94±0.06	1.54±0.14	1.68±0.13
48	2.01±0.03	2.05±0.02	1.17±0.04	1.14±0.04	0.71±0.07	0.64±0.05	0.88±0.04	0.88±0.04	2.09±0.15	2.13±0.09
	Creatinine (µg/dl)		BUN [$\log_{10}$(mg/dl)]		Na (mmol/L)		K (mmol/L)		Cl (mmol/L)	
4	477±31	457±30	1.08±0.02	1.05±0.02	139±1	139±1	3.7±0.2	3.4±0.1	112±1	113±1
24	459±90	463±48	1.23±0.09	1.29±0.07	139±1	140±1	3.5±0.1	3.5±0.1	114±1	114±1
48	503±131	539±34	1.29±0.11	1.24±0.03	139±1	140±1	3.7±0.1	3.7±0.1	113±1	114±1
	pH		PCO_2 (mmHg)		HCO_3 (mmol/L)		Lactate [log(mmol/L)]		PO_2(mmHg)	
4	7.48±0.01	7.49±0.01	23.9±1.0	25.7±1.1	18.3±0.8	19.7±0.8	0.35±0.05	0.18±0.04	111.7±2.4	106.6±2.6
24	7.47±0.02	7.49±0.01	25.6±1.5	25.4±1.2	18.8±1.0	19.5±0.7	0.13±0.08	0.20±0.06	110.7±6.9	111.0±3.5
48	7.49±0.01	7.47±0.01	28.8±1.5	30.2±1.0	22.1±1.0	22.1±0.8	-0.01±0.05	-0.10±0.05	105.3±5.3	109.2±22.1

Na – plasma sodium; K – plasma potassium; Cl – plasma chloride; pH – arterial pH; PCO_2 – arterial carbon dioxide; HCO_3 – bicarbonate; WBC – total white blood cell count
